# Turkish translation, validity, and reliability of the European organization for research and treatment of cancer quality of life-high dose chemotherapy29 in patients undergone hematopoietic stem cell transplantation

**DOI:** 10.55730/1300-0144.6001

**Published:** 2025-04-17

**Authors:** Vesile YILDIZ KABAK, Sevilay KARAHAN, Elifcan ALADAĞ KARAKULAK, Tülin DÜGER, Songül ATASAVUN UYSAL, Fulya İPEK ERDEM, Hakan GÖKER

**Affiliations:** 1 Department of Basic Physiotherapy and Rehabilitation, Faculty of Physical Therapy and Rehabilitation, Hacettepe University, Ankara, Turkiye; 2 Division of Biostatistics, Department of Basic Sciences, Faculty of Medicine, Hacettepe University, Ankara, Turkiye; 3 Division of Hematology, Department of Internal Medicine, Faculty of Medicine, Hacettepe University, Ankara Turkiye; 4 Faculty of Physical Therapy and Rehabilitation, Dokuz Eylül University, İzmir, Turkiye

**Keywords:** Hematopoietic stem cell transplantation, quality of life, reliability, validity

## Abstract

**Background/aim:**

Our aim was to investigate psychometric properties of the Turkish version of the European organization for research and treatment of cancer quality of life-high dose chemotherapy29 (EORTC QLQ-HDC29) in patients treated with hematopoietic stem cell transplantation (HSCT).

**Materials and methods:**

Patients between the ages of 18 and 65 years and undergone HSCT were included. The European organization for research and treatment of cancer quality of life questionnaire–cancer30 the Eastern cooperative oncology group performance score, and the functional assessment of cancer therapy–bone marrow transplant were used to determine convergent validity. Reliability was assessed through the calculation of Cronbach’s alpha and intra-class correlation coefficient (ICC) values.

**Results:**

Totally 151 patients were included. The convergent validity analysis between the EORTC QLQ-HDC29 and the other variables revealed significant, low to strong correlations (p < 0.05). The test-retest reliability of the questionnaire was excellent (ICC values ranged from 0.886 to 1.000). The internal consistency values were acceptable except for the “Worries/Anxiety” and “Inpatient Issues” scales (ranged from 0.175 to 0.985).

**Conclusion:**

The reliability and validity of Turkish version of the EORTC QLQ-HDC29 was feasible and acceptable in patients treated with HSCT. Further research is required to ascertain the psychometric properties of the EORTC QLQ-HD29.

## 1. Introduction

High-dose chemotherapy (HDC) followed by hematopoietic stem cell transplantation (HSCT) has been widely used in patients diagnosed with diseases that affect bone marrow [1]. According to the European group for blood and marrow transplantation current reports, more than 700,000 patients received HSCT to date and more than 40,000 new patients per year are prescribed to undergo HSCT [2].

The physical, psychological, social, sexual, and vocational components of a patient’s quality of life (QOL) are all adversely affected by transplantation procedures [3–5]. These diverse issues have resulted in the development of several side effects. For this reason, each of these symptoms and their reflections on an individual’s QOL should be examined in detailed and comprehensively [6,7]. Disease- and condition-specific questionnaires have been developed to meet this need [8,9]. The European organization for research and treatment of cancer quality of life-high dose chemotherapy29 (EORTC QLQ-HDC29) is one of the questionnaire to measure QOL level specifically in patients undergoing HSCT [6]. The number of surveys tailored to patients treated with HSCT is very limited in the literature.

The EORTC QLQ-HDC29 is originally developed in English, German, Norwegians, French, and Italian [6]. It was translated and validated in Korean [10], and Icelandic [11], and there is no translation of the questionnaire in Turkish. Since cultural differences may alter the interpretation of the survey items, we aimed to investigate the psychometric properties of the Turkish version of the EORTC QLQ-HDC29.

## 2. Material and methods

The present study was performed at Hacettepe University Oncology Hospital in Türkiye. Data was collected while patients were in the hospital for HSCT or during their regular post-HSCT hospital visits. Patients undergone HSCT at Hacettepe University were included in the present study. Inclusion criteria included being ages between 18 to 65 years, who have treated with HSCT and are still hospitalized or outpatient, and being able to cooperate. Patients willing to participate in the study was signed the consent form and included in the present study. Hacettepe University Non-Interventional Clinical Trials ethical committee approved the study protocol.

### 2.1. Translation of the EORTC QLQ-HDC20 English form into Turkish form

Firstly, permission from the EORTC team to develop a Turkish form of the EORTC QLQ-HDC29 was received. The EORTC translation manual was followed during the translation process [12]. Two native Turkish researchers (VYK, FI) independently translated the EORTC QLQ-HDC29 English form to Turkish. If disagreements were detected, an independent senior researcher (TD) resolved them during the reconciliation process. Following this process, a backward translation was performed by two independent researchers who have a very good command of Turkish and English. The results of these steps (2 forward translations, reconciliation, and 2 backward translations with comments) were sent to the EORTC team to review the process. The comments of an external proofreader were received from the EORTC organization. According to the comments, the form reviewed and all issues had been resolved. When all of the stages were performed, the pilot testing including 10 patients was performed for the linguistic validation. During the pilot testing, there was no problem with understanding the items. After the pilot testing, a report including the patients’ comments regarding the Turkish form of the EORTC QLQ-HDC29 was sent to the EORTC team and they reviewed the comments and finalized the process.

### 2.2. Recorded outcomes

To perform Turkish reliability and validity of the EORTC QLQ-HDC29, some data regarding demographic and medical variables, performance level, and QOL level of the participants were collected. These parameters are mentioned below.

#### 2.2.1. Demographic and medical data

Demographic data including patients’ age, sex, body mass index, educational level, and marital status were recorded. Medical data including diagnosis, time since diagnosis, HSCT type, time since HSCT, the presence of comorbidity, and HSCT-related complications were recorded.

#### 2.2.2. The European organization for research and treatment of cancer quality of life-high dose chemotherapy (EORTC QLQ-HDC29)

The EORTC QLQ-HDC29 is a patient-reported outcome measure that tailored to assess HSCT-related problems [6]. It consists of 6 scales named Gastro-Intestinal Side Effects, “Worries/Anxiety”, “Inpatients Issues”, “Body Image, Impact on Family, and Sexuality, and 8 single items named “Skin Problems”, “Fever”, “Urine Frequency”, “Aches in Bones”, “Finishing Things”, “Spirituality”, “Fertility”, and “Regular Drug”. The items are scored between 1 to 4 points. A higher score represents a higher symptom burden, except for the “Spirituality” item which is scored the opposite.

#### 2.2.3. Performance level

Patients’ performance level was recorded by using the Eastern Cooperative Oncology Group Performance Score (ECOG). The ECOG categorizes the patients according to the general daily activities in 5 groups. While getting “0 points” from the ECOG means patients who are active with no restriction in any activity, getting “4 points” means patients who are completely dependent in bed [13].

#### 2.2.4. The European organization for research and treatment of cancer quality of life questionnaire–cancer30 (EORTC QLQ-C30)

The European organization for research and treatment of cancer quality of life questionnaire–cancer30 (EORTC QLQ-C30 was used to measure QOL of participants. The EORTC QLQ-C30 was designed to measure the QOL level of cancer patients. It comprises of 30 items are scored 0 points (not at all) to 4 points (too much). The total score of the EORTC QLQ-C30 is calculated into three subtitles: general health score, functionality score, and symptom score. While a higher score represents better QOL in general and functionality; higher symptom scores represent higher symptom burden in patients [14,15].

#### 2.2.5. Functional assessment of cancer therapy–bone marrow transplant (FACT-BMT)

The functional assessment of cancer therapy–bone marrow transplant (FACT-BMT) is an internationally validated and HSCT-specific questionnaire that assesses different aspects of treatment. It included 37 items and two parts: Functional assessment of cancer treatment-general version (FACT-G) and bone marrow transplant subscale (BMTS). The total score ranges from 0 to 196 points and a higher score indicates better QOL [16,17].

### 2.3. Statistical analysis

The IBM SPSS Statistics 23 Version was used to perform statistical analysis. Number (n) and percentage (%) values for qualitative data, and mean (χ̄), standard deviation (SD), and minimum–maximum values for quantitative data were given. Type I error was set at 0.05 for all statistical analyses. The Cronbach’s alpha was calculated to determine internal consistency. To determine test–retest reliability, the intra-class correlation coefficients (ICCs) were calculated. The Kolmogorov–Smirnov test was used to determine normality distribution and the data showed a normal distribution. Pearson’s correlations between the EORTC-HDC29, the EORTC QLQ-C30, the FACT-BMT, and the ECOG performance level were calculated to determine the convergent validity.

## 3. Results

The present study included 151 patients of whom 60% of participants were male. The demographic and medical characteristics of the participants were showed in Table 1. The most prevalent diagnosis was multiple myeloma followed by Hodgkin lymphoma and acute myeloid leukemia in the participants. Autologous HSCT was mostly preferred in patients. The mean duration since diagnosis was nearly 3 years and the mean time after HSCT was more than one year. Comorbidity and HSCT-related complication rates were low and patients had mostly good performance levels measured by the ECOG.

Descriptive statistics and the psychometric properties of the EORTC QLQ-HDC29 were showed in Table 2. Cronbach alpha values of the EORTC QLQ-HDC29 subtitles ranged from 0.175 to 0.985. The ICC scores were found between 0.886 and 1.000. There was no significant difference in EORTC QLQ-HDC29 scores in participants according to the presence of comorbidity and HSCT type. When the results were compared according to sex, the women had higher symptom burden than men in the “Worries/Anxiety”” scale (23.90 ± 18.75 vs. 10.00 ± 15.19 points; p = 0.006) and they got higher scores in the “Spirituality” scale (25.13 ± 27.65 vs. 13.85 ± 19.98 points; p = 0.009). Correlation analysis were presented in Table 3. Our results revealed significant low to strong associations between the EORTC QLQ-HDC29 subtitles and the EORTC QLQ-C30, the FACT-BMT, and the ECOG scores (p < 0.05).

## 4. Discussion

Developing culture-specific surveys or adapting an existing survey in a language can increases the usability of that survey and helps us better understand the problems of individuals in that society. In our study, the psychometric properties of the EORTC QLQ-HDC29 questionnaire were investigated by translating it into Turkish. Our results showed that the Turkish version of the EORTC QLQ-HDC29 has acceptable reliability and validity. The internal consistency of the items was suitable except for the “Worries/Anxiety” and the “Inpatient Issues” scales.

We included patients undergone autologous and allogeneic HSCT in the present study to ensure the usability of the questionnaire in all patients receiving this treatment. In addition, both inpatient and outpatient individuals undergone HSCT were included. We included patients with different diagnoses and performance level, and the most prevalent diagnosis was multiple myeloma followed by Hodgkin lymphoma and acute myeloid leukemia.

During the translation phase of the EORTC QLQ-HDC29, there was no need to modify any item of the questionnaire to culturally adapt. In pilot testing of the draft version of the translated form, patients reported that each item was understood and they did not experience any difficulty. Regarding items (28 and 29) asking about sexual life, some patients (n = 63) reported not being willing to answer. Sexual life is a cultural taboo in our country [18], which we experienced in our previous study [17]. Despite the low participation, the internal consistency of this scale was acceptable in our study.

Convergent validity analysis of the EORTC QLQ-HDC29 revealed acceptable correlation coefficients with the EORTC QLQ-C30, the ECOG, and the FACT-BMT. We determined that the correlation coefficient values between the EORTC QLQ-HDC29 and the FACT-BMT were higher than the other questionnaires. This result could be derived from the questionnaires specific to HSCT were more closely associated than the others. The correlation coefficient values were low to strong which is similar with a previous study [10]. As suggested previously, HSCT-specific questionnaires may be more appropriate for evaluating QOL in this cohort than generic cancer questionnaires [19].

We found acceptable reliability, which is analyzed with internal consistency, in most scales of the EORTC QLQ-HDC29 except for the scales: “Worries/Anxiety” and “Inpatient Issues”. The level of internal consistency of the “Inpatient Issues” scale was also low in the Korean version of the EORTC QLQ-HDC29 [10]. Moreover, the internal consistency of the “Impact on Family” scale was found poor in another previous study [20]. Regarding the “Inpatient Issues” scale, the items were filled by only patients who were hospitalized in the present study. The small number of patients (n = 37) may decrease the internal consistency of the scale. The validity of the “Worries/Anxiety” scale was also not satisfactory in our study, contrary to the Korean and original versions in which moderate validity was found for the scale [10]. Since there are some inconsistencies regarding the internal consistency results, we suggest that the psychometric properties of the EORTC QLQ-HDC29 should be further investigated in different languages.

Test and retest reliability of all scales and single items of the EORTC QLQ-HDC29 were excellent in our study. To our knowledge, there is no study investigating the reliability of the EORTC QLQ-HDC29. We suggest that the other reliability analysis in different languages may be investigated in future studies. It is valuable that the responsiveness of a questionnaire to the different variables such as sex, HSCT-type, existing diseases, and other parameters. Our results showed that the Turkish form of the EORTC QLQ-HDC29 was sensible to sex. Women had a higher symptom burden than men in the “Worries/Anxiety” scale, while their “Spirituality” score was higher than men. Sex differences especially regarding anxiety and depression were investigated previously, and women have been found more emotional symptom burden than man diagnosed with cancer [21,22]. This might have resulted from the fact that women were more responsible for household chores and child care than males in Turkish culture [23]. Having more responsibilities may result with more emotional distress in women patients.

We have some limitations in the present study. First of all, the included patients in the present study were recruited from only one center. This may negatively affect the generalizability of our results. However, this center where patients are recruited, is one of the largest hospital in our country where patients from different cities apply for HSCT. Secondly, since our study design was cross-sectional, the longitudinal changes of the EORTC QLQ-HDC29 scores could not be measured in the present study. Future studies may further investigate the longitudinal changes and the psychometric properties of the EORTC QLQ-HDC29. Lastly, the responsiveness of the EORTC QLQ-HDC29 to various complications especially Graft-versus Host disease associated with allogeneic HSCT did not investigated in the present study. Sub-group analysis was not feasible due to the small number of participants in the current study who experienced HSCT-related complications.

In conclusion, the Turkish version of the EORTC QLQ-HDC29 has excellent reliability scores, with the exception of the “Worries/Anxiety” and “Inpatient Issues” scales, which had poor internal consistency. The convergent validity of the EORTC QLQ-HDC29 was excellent. Our results suggest that the Turkish form of the EORTC QLQ-HDC29 was valid and the psychometric properties of the questionnaire might be further investigated in different languages.

## Figures and Tables

**Table 1:** Demographic and medical characteristics of the participants (n = 151).

Recorded variables	Mean ± SD (min-max)
Age, years	50.04 ± 12.08 (20–70)
Body mass index, kg/m^2^	26.65 ± 4.64 (16.41–45.78)
Duration since diagnosis, month	31.54 ± 17.25 (3–84)
Duration since HSCT, month	15.67 ± 9.84 (1–48)
Sex, n (%)	
Female	61 (40.40)
Male	90 (59.60)
Educational status, n (%)	
Illiterate	6 (4.0)
Primary school	48 (31.8)
Secondary school	14 (9.3)
High school	40 (26.5)
Graduate	40 (26.5)
Postgraduate	3 (2.0)
Marital status, n (%)	
Married	133 (88.1)
Single	18 (11.9)
The presence of comorbidity, n (%)	
Yes	30 (19.9)
No	121 (80.1)
Comorbidities, n (%)	
Cardiovascular disorder	23 (15.2)
Chronical kidney disease	2 (1.3)
Pulmonary disease	2 (1.3)
Other	3 (2.0)
Diagnosis, n (%)	
Multiple myeloma	56 (37.1)
Hodgkin lymphoma	31 (20.5)
Acute myeloid leukemia	27 (17.9)
Non-Hodgkin lymphoma	18 (11.9)
Acute lymphoblastic leukemia	15 (9.9)
Myelodysplastic syndrome	4 (2.6)
ECOG performance score, n (%)	
0: Active	25 (16.6)
1: Limited vigorous physical activity	88 (58.3)
2: Symptomatic, independent in ADL	34 (22.5)
3: Limited in ADL, dependent in bed more than 50% of time	4 (2.6)
Transplantation type, n (%)	
Autologous	104 (68.9)
Allogeneic	47 (31.2)
HSCT-related complication n (%)	
Yes	37 (24.5)
No	114 (75.4)

HSCT: Hematopoietic stem cell transplantation, ADL: Activities of daily life, ECOG: Eastern Cooperative Oncology Group

**Table 2:** Descriptive statistics and the psychometric proporties of the EORTC QLQ-HDC29.

EORTC QLQ-HDC29	Mean ± SD	Min-Max	Cronbach alpha	ICC (95% Confidence Interval)
Gastro-Intestinal Side Effects Scale	10.59 ± 14.97	0.00–66.67	0.807	0.990 (0.983–0.994)
Worries/Anxiety Scale	19.10 ± 17.06	0.00–91.67	0.545	0.953 (0.925–0.971)
Inpatient Issues Scale	38.73 ± 10.99	22.22–55.56	0.175	1.000 (1.000–1.000)
Body Image Scale	9.49 ± 19.57	0.00–100.00	0.771	0.992 (0.987–0.995)
Impact on Family Scale	12.05 ± 17.79	0.00–83.33	0.805	0.998 (0.997–0.999)
Sexuality Scale	36.45 ± 35.27	0.00–100.00	0.985	0.992 (0.983–0.996)
Single-item Scales				
Skin Problems	12.14 ± 19.04	0.00–66.67	NA	1.000 (1.000–1.000)
Fever	7.77 ± 18.28	0.00–100.00	NA	1.000 (1.000–1.000)
Urine Frequency	11.47 ± 20.38	0.00–100.00	NA	0.982 (0.971–0.989)
Aches in Bones	18.54 ± 21.98	0.00–100.00	NA	0.980 (0.968–0.988)
Finish Things	9.49 ± 18.61	0.00–66.67	NA	0.886 (0.821–0.928)
Spiritual	18.44 ± 23.97	0.00–100.00	NA	0.981 (0.970–0.988)
Fertility	5.55 ± 19.24	0.00–66.67	NA	1.000 (1.000–1.000)
Regular Drugs	13.06 ± 22.88	0.00–100.00	NA	0.983 (0.973–0.990)

EORTC QLQ-HDC29: The European Organization for Research and Treatment of Cancer Quality of Life-High Dose Chemotherapy29, ICC: Intra-Class Correlation.

**Table 3:** Correlations between EORTC QLQ-HDC29 scales and the other recorded variables.

EORTC QLQ-HDC29	EORTC QLQ-C30			ECOG	FACT-BMT Total Score
	General health	Function	Symptom		
GISE Scale	−0.168^*^	−0.171^*^	0.297^**^	0.234^**^	−0.497^**^
Worries/Anxiety Scale	−0.297^**^	−0.376^**^	0.299^**^	0.211^**^	−0.592^**^
Inpatient Issues Scale	0.211^*^	0.199	−0.075	−0.142	−0.256^*^
Body Image Scale	−0.044	−0.092	0.115	0.165^*^	−0.619^**^
Impact on Family Scale	−0.246^**^	−0.230^**^	0.242^**^	0.308^**^	−0.655^**^
Sexuality Scale	−0.239	−0.123	0.205	0.197	−0.606^**^
Skin Problems	−0.160	−0.211^*^	0.322^**^	0.281^**^	−0.260^*^
Fever	−0.005	0.069	−0.219^*^	0.057	−0.250^*^
Urine Frequency	0.010	−0.071	0.138	0.065	−0.293^*^
Aches in Bones	−0.218^*^	−0.183	0.430^**^	0.240^**^	−0.260^*^
Finishing Things	−0.228^**^	−0.248^**^	0.379^**^	0.294^**^	−0.423^**^
Spiritual	−0.109	−0.245^**^	0.232^**^	0.148	−0.308^**^
Fertility	−0.482^*^	−0.419^*^	0.394	0.234	−0.054
Regular Drugs	−0.210^*^	−0.135	0.259^**^	0.342^**^	−0.568^**^

EORTC QLQ-HDC29: The European Organization for Research and Treatment of Cancer Quality of Life-High Dose Chemotherapy29, EORTC QLQ-C30: European Cancer Organization for Research and Treatment of Cancer Quality of Life Questionnaire–Cancer30, ECOG: Eastern Cooperative Oncology Group, FACT-BMT: Functional Assessment of Cancer Therapy–Bone Marrow Transplant, GISE: Gastro-Intestinal Side Effect.
